# Editorial: Recent advances in cellulosomes and their application in bioenergy production

**DOI:** 10.3389/fmicb.2025.1720268

**Published:** 2025-10-23

**Authors:** Marimuthu Anandharaj, Jui-Jen Chang, Yannick J. Bomble

**Affiliations:** ^1^U.S. Department of Energy Joint Genome Institute, Lawrence Berkeley National Laboratory, Berkeley, CA, United States; ^2^Department of Medical Research, China Medical University Hospital, China Medical University, Taichung, Taiwan; ^3^Graduate Institute of Integrated Medicine, China Medical University, Taichung, Taiwan; ^4^Biosciences Center, National Renewable Energy Laboratory, Golden, CO, United States

**Keywords:** cellulosome, protein complex, biofuel production, enzyme engineering, biodegradation, consolidated bioprocessing, sustainable energy, co-culture

## 1 Introduction

Lignocellulosic biomass represents an abundant feedstock to produce valuable products such as biofuels and biochemicals. However, the deconstruction of plant biomass into simple sugars is a rate-limiting step due to its inherent recalcitrance and the costs associated with its pre-treatment. Interestingly, certain cellulolytic microorganisms have evolved to efficiently solubilize and utilize lignocellulosic biomass through the synergistic action of unique catalytic enzymes. One of the most notable examples of such a system is the “cellulosome,” an enzymatic complex produced by some anaerobic bacteria and fungi (e.g., *Clostridium thermocellum*) that can effectively break down plant cell walls into simple sugars, which can then be used to produce value-added products. Recent advances in synthetic biology have enabled the engineering of cellulosomes and enhancement of their activity and stability, including the incorporation of new types of enzymes and designer scaffoldins into these cellulosomes. Additionally, synthetic biology tools have enabled the creation of tailor-made cellulosomes (also called designer cellulosomes) for specific feedstocks, thus further improving their efficiency and reducing the cost of bioenergy production.

However, there are still many challenges to be overcome, such as optimizing the production and application of cellulosomes at an industrial scale and developing systems to better control the composition of cellulosomes. Hence, the major goal of this Research Topic is to gather novel research on the isolation, identification of novel cellulosome-producing bacterial or fungal species from various habitats, the identification and characterization of novel cellulosomal enzymes to achieve efficient biomass degradation, and the optimization of processes using cellulosome-producing bacteria in a consolidated bioprocessing context. Within this Research Topic, four articles were published, which include two research articles and two review articles.

## 2 Overview of contributions

Minor et al. performed a large-scale genome-wide survey of over 305,693 sequenced bacterial genomes to identify novel cellulosome-producing bacteria. They narrowed down their searches to glycoside hydrolases (DocGHs), which contain dockerins. They also extended their search to find scaffoldins (that contain cohesins that dockerins bind to form cellulosomes). Based on their research, they have identified 33 bacterial species able to produce cellulosomes, among them 10 novel bacterial species, including *Acetivibrio mesophilus, Clostridium cibarium, Clostridium pasteurianum* etc. This study significantly expands our knowledge on the diversity of major cellulosome-producing bacteria. Similarly, comparative genomic and phylogenetic analysis revealed a distinct organization of cellulosomes among different genera of cellulosome-producing bacteria. For example, *Acetivibrio* and *Ruminococcus* species possess complex cellulosomes with a high diversity of scaffoldins and dockerin-fused glycoside hydrolase enzymes, which can efficiently degrade lignocellulosic plant biomass. In contrast, they found that *Ruminiclostridium* and *Clostridium* lineages display relatively simple but functionally diverse cellulosome complexes. These findings can help us understand cellulosome complexes across different bacterial species and develop/engineer novel designer cellulosomes for efficient degradation of plant materials for the sustainable production of biofuels, renewable chemicals, and biomaterials.

The review article authored by Hsin et al. provides a comprehensive knowledge of both bacterial and fungal cellulosomes. They extensively discuss the unique structural organization and functions of fungal cellulosomes and how they differ from their bacterial counterparts. Moreover, this review examines how bacterial cellulosomes achieve remarkable efficiency in biomass degradation, and how others have adapted this knowledge to improve the structural stability of “designer cellulosome.” Most importantly, to achieve the complete lignocellulosic biomass degradation on an industrial scale, they highlight three major focus areas, including the discovery of novel lignocellulolytic bacterial or fungal species and their enzymes, bioengineering cellulosomal enzymes to improve their thermostability, and structural optimization of synthetic cellulosome complexes. By combining microbial diversity with protein engineering and systems-level design, this review presents a roadmap for overcoming the current bottlenecks in lignocellulosic biomass to biofuel conversion.

Similarly, Lindič and Vodovnik provide an in-depth analysis of the molecular architecture and dynamic mechanisms that support cellulosome functions. This review highlights the significance of carbohydrate-binding modules (CBMs) on substrate recognition and binding to various substrates. Interestingly, the authors found that the X2 modules, whose exact function is essentially unknown, support the functions of CBM and increase the scaffoldin stability. They also found that the engineering of CBMs can enhance their substrate specificity and binding efficiency. Cohesin-dockerin interactions play a vital role in the assembly of cellulosomal components and the type and species specificity of these interactions can be utilized to develop a customized synthetic cellulosome complex with enhanced enzyme synergism. This review bridges the fundamental structural biology with applied biotechnology to provide an in depth understanding of these natural nanomachines, master of plant cell wall deconstruction, that are cellulosomes.

Nhim et al. performed a comparative study to demonstrate the factors that are essential to enable high solids fermentation in a consolidated bioprocessing (CBP) context with *Clostridium thermocellum*. In this study, they evaluate the importance of the presence of a co-culture partner to relieve cellobiose inhibition to the cellulosomes of *C. thermocellum*. This coculture shows improved performance with the *Thermobrachium celere* strain producing a key beta-glucosidase. Additionally, the authors demonstrate that non-productive binding to lignin or other structures that arise during fermentation can be alleviated with the use of a surfactant, tween 20, which improves enzymatic performance and therefore increases the amount of accumulated glucose and overall levels of solubilization. Finally, they demonstrate the advantages of a semi fed batch approach to circumvent common drawbacks with high solids fermentation in CBP. Taken together these results help to further improve our understanding of CBP at high solids and pave the way for better performance in the production of sustainable biofuel and biochemical.

## 3 Conclusion

Cellulosomes are incredible nanomachines that have evolved to efficiently solubilize lignocellulose. The understanding of these enzymatic systems, their composition, activity, and dynamics has drastically improved since first identified by Bayer and Lamed in the early 80s. However, there is still much to study and discover to fully leverage the potential of these cellulolytic complexes. Recent advancements in synthetic biology and molecular biology techniques have enabled the designer cellulosome components for enhanced substrate specificity, catalytic efficiency, and stability. The articles included in this Research Topic represent another step in this direction.

